# Effect of maternal prenatal food supplementation, gestational weight gain, and breast-feeding on infant growth during the first 24 months of life in rural Vietnam

**DOI:** 10.1371/journal.pone.0233671

**Published:** 2020-06-25

**Authors:** Phi N. Quyen, Hoang T. Nga, Benjamin Chaffee, Tu Ngu, Janet C. King

**Affiliations:** 1 National Institute of Nutrition, Hanoi, Vietnam; 2 University of California San Francisco, San Francisco, California, United States of America; 3 Children’s Hospital Oakland Research Institute, Oakland, California, United States of America; Institut de recherche pour le developpement, FRANCE

## Abstract

Growth faltering among children during the first five years of life is a common problem among low and middle-income countries. The purpose of this study was to determine the effect of a nutrient-rich, food-based supplement given to Vietnamese rural women prior to and/or during pregnancy on the growth of their infants during first 24 months of life and to identify maternal and newborn factors associated with the infant’s growth. This prospective cohort study included 236 infants born to mothers who had received nutritional advice or a food supplement from pre-conception to term or from mid-gestation to term as part of a prior randomized controlled trial. Infant anthropometry and feeding information were monitored monthly and the infant weight for age Z-score (WAZ), length for age Z-score (LAZ), and weight for length Z-score (WLZ) were assessed at 6, 12, 18, and 24 months of age using mixed-effects regression modeling. Compared to the non-supplemented mothers, infants born to mothers receiving food supplementation from mid-gestation to term had significantly higher WLZ only at 18 months (p = 0.03) and did not differ in other outcomes. Supplementation from pre-conception to term did not affect infant growth at any time point during the first 24 months. In the entire study cohort, maternal height and gestational weight gain were positively associated with the infant’s WAZ and LAZ from 6 to 24 months of age. Programs designed to improve gestational weight gain among women performing demanding physical work throughout a reproductive cycle may improve postnatal infant growth.

**Trial registration**: Registered Clinical Trials.Gov: NCT01235767.

## Introduction

Childhood stunting, defined as a length for age Z-score below 2 standard deviations of the mean, is a prevalent problem among infants and young children living in lower-income households. Worldwide, about 162 million children under 5 years of age are stunted, and all but 2 million of those children live in low- or middle-income countries (UNICEF & World Bank estimates, 2013). In Vietnam, about one-third, or 1.9 million, of the children under 5 years of age are stunted, and the stunting rates are three times higher in low-income households and in ethnic minority groups (http://unicefvietnam.blogspot.com/2015/01/tackling-malnutrtion-and-stunting-in.html). Poor quality diets that are watered down and have low levels of energy and nutrients contribute to the growth faltering. Linear growth faltering often becomes evident after 6 months of age when cereal gruels with low nutrient densities replace breast milk or infant formula.

Several groups have studied the impact of maternal nutrient supplementation during pregnancy on the subsequent growth of infants in low-income countries [1–6]. The results are diverse. Two groups failed to find a positive effect of a lipid-based nutrient supplement (LNS) taken either during pregnancy [3] or during pregnancy and six month postpartum [1] on growth during the first year or two of life. However, several other studies showed a positive effect of maternal supplementation on child growth over the first five years of life [2, 5, 6]. For example, supplementation of the mother during pregnancy with the UNIMMAP preparation containing 15 micronutrients improved the infant’s weight for length and head circumference at 12 months of age compared to supplementation with only iron and folic acid [2]. This suggests that supplementation with a broad array of micronutrients has a greater impact on infant growth than supplementation with only several nutrients.

Maternal gestational weight gain is another important determinant of postnatal growth in higher and lower income countries [7]. A higher gestational weight gain, particularly in lower income countries such as Vietnam, may reflect a lower level of physical work during pregnancy and, therefore, better fetal nourishment. However, in higher income countries, a high gestational weight gain combined with a high pre-pregnancy BMI significantly increased the infant’s weight/height percentile at 12 months of age [8]. The relationship between gestational weight gain and subsequent infant growth in underweight women is less well understood. A recent study of Chinese women and their offspring showed that the maternal BMI and weight gain were positively associated with the weight-for-age Z-score (WAZ) and the length-for-age Z-score (LAZ) [9]. Thus, we proposed that a higher gestational weight gain in underweight, farming Vietnamese women could improve the infant’s postnatal growth.

Although most recent studies of maternal nutrition and pregnancy outcomes have focused on micronutrient supplementation [10, 11], maternal energy and protein intakes also play essential roles in fetal and neonatal growth [12–14]. In 1980, Professor Tu Giay recognized that improved nutrition was essential for improving the health of the Vietnamese population [15]. Thus, he founded the Vietnam National Institute of Nutrition to train future leaders in medicine, food, and nutrition. His work also provided the basis for a novel agricultural program, called the VAC, (Vuon, Ao, Chuong in Vietnamese, meaning garden, pond, livestock pen), system that supported the local production of fish, pork, poultry, eggs, and vegetables by the farmers.

The objective of this present study was to determine the effect maternal and infant nutritional factors on infant growth during the first 24-months of life. Included in this assessment is the consumption of a small nutrient-dense food-based supplement based on VAC foods prior to and/or during pregnancy, which had been part of a prior randomized controlled trial. The impact of the food-based supplement on pregnancy outcomes are summarized in a separate paper [16].

## Materials and methods

The study was conducted in 29 communes of the Cam Khe district in the Phu Tho province, Vietnam. The site is located approximately 100 km Northwest of Hanoi, and 90% of its inhabitants live in rural areas. As is typical in rural Vietnam, a health center in each commune provides prenatal and infant health care. The original trial, fully described elsewhere [16], was conducted over a four-year period, 2011–2015. Non-pregnant women were recruited for the study when they registered to marry.

After informed consent was obtained, the women were randomly assigned to one of the three interventions using the random number generator in Excel (Microsoft, Redmond, WA, USA): 1) the food-based supplement consumed 5 days/week from study recruitment to term (approximately 11 months); 2) the food-based supplement consumed 5 days/week from 16 weeks of gestation to term (approximately 5 months), or 3) routine prenatal care without the supplementation. The supplement meals were prepared daily according to a 10-day cycle of menus based on local animal-source foods (including either pork, shrimp, liver, blood, or embryonated duck eggs) and dark-green leafy vegetables. Health workers provided the meals to the women 5 days/week at a designated eating site. Meal consumption was observed, and the quantity consumed was recorded by the health workers.

At entry to the study, a medical doctor at the commune health center completed an evaluation of maternal socio-demographic and anthropometrical characteristics. A blood sample was collected, and anthropometric measurements were made. The usual dietary intakes of the women were assessed from two repeated non-consecutive 24-hr recalls. These maternal measurements were repeated at mid (16 weeks) and late (32 weeks) gestation and are reported elsewhere [16]. The Communal Health Station Director contacted the participants twice a month for a morbidity interview and to determine their menstruation status. These twice-monthly maternal assessments were discontinued after delivery.

### Ethics approval

The study was reviewed and approved by the Ethical and Scientific Committees of the National Institute of Nutrition, Hanoi, Vietnam, and by the Institutional Review Board of the Children’s Hospital Oakland Research Institute. Informed consents were obtained from each of woman when she was enrolled in the trial prior to conception. At that time, the mothers consented to have their infants studied for the first 24 months of life.

### Infant outcome measurements

Birth measurements were taken at the hospital and again at the subject’s home within 7 days of delivery. Gestational age was estimated using two procedures: 1) an ultrasound measurement using a Mindray DP-1100 Plus portable ultrasound instrument (Shenzhen Mindray Bio-Medical Electronics, China) and, 2) by the number of weeks from the last menstrual period (LMP) recorded in the participant’s logbook, checked twice monthly at the Health Station, and the date of delivery. Pre-term birth was defined as a birth before 37 weeks of gestation, and small for gestational age (SGA) was defined as a birth weight < 10^th^ percentile for gestational age, based on the INTERGROWTH-21st Project [17]. The infant’s food intake was assessed from two 24-hour recalls with the Mother every three months and an assessment of infant feeding practices and anthropometry.

Infants’ weight, length, head circumference (HC), and mid-upper-arm circumference (MUAC) were measured at birth and every 3 months of age thereafter. Trained staff made all of the measurements using standardized procedures and equipment (i.e., infant scales, length measuring board, non-stretch tape, forms, and questionnaires). The body weight of the naked infant was measured to the nearest 5 g using the Laica PS3001W electronic infant scale (LAICA SpA, 36020 Ponte di Barbarano, Vicenza, Italy, www.laica.it). Recumbent length was measured heel to crown (without shoes and with toes pointing directly upward and keeping the knees straight) to the nearest mm using a portable infantometer (Seca 417). Head circumference was measured with a narrow, flexible, non-stretchable tape (Seca 212) just above the supraorbital ridges over the most prominent part of the frontal bulge and the occiput. Mid upper-arm circumference was measured with a narrow, flexible, non-stretchable tape (Seca 212) at the mid-point of the right upper arm, between the acromion process and the tip of the olecranon. Duplicate anthropometric measurements were measured to the nearest mm and averaged [18]. A third measurement was performed if the initial two values differed by more than 10%. When that was done, the average of the two closest measurements was used. The field coordinator regularly monitored the anthropometric measurements and repeated the measurements on a number of infants to confirm accuracy. Scales, length-measuring boards, and non-stretchable measuring tape were checked before all measurements were made to assure that they were in good quality.

Weight-for-age Z-score (WAZ), length-for-age Z-score (LAZ), and weight-for-length Z-score (WLZ) were calculated using a sex-specific reference database from the World Health Organization (Anthro 3.2.2 software [19]). Underweight, stunting and wasting were defined as WAZ, LAZ and WLZ as >2SD below the WHO Child Growth Standards [20, 21].

Information regarding infant feeding practices (i.e., duration of exclusive breast feeding, introduction of complementary drinks and solid foods, the amount and content of homemade complementary foods consumed, and the intake of micronutrient supplements) were collected at each of the four postpartum visits scheduled every 6 months using a standardized questionnaire. Specifically, the mother was asked how frequently the child was breastfed each day, at what age breastfeeding was discontinued, when solid foods were introduced into the child’s diet, and what vitamin and mineral supplements were consumed. We defined "exclusive breastfeeding" as no other food or drink, including water, except breast milk and the use of Oral Rehydration Solution, if it was prescribed, and drops or syrups containing vitamins, minerals, and medicines.

### Sample size

A total of 460 women consented to participate in the original study and were assigned to one of the following three groups.

Group PC-T: ASF supplement + leafy greens from Pre-Conception to Term, N = 150.

Group MG-T: ASF supplement + leafy greens from Mid-Gestation (about 16 weeks) to Term, N = 153.

Group RPC: Routine prenatal care, N = 157.

**Fig 1:**
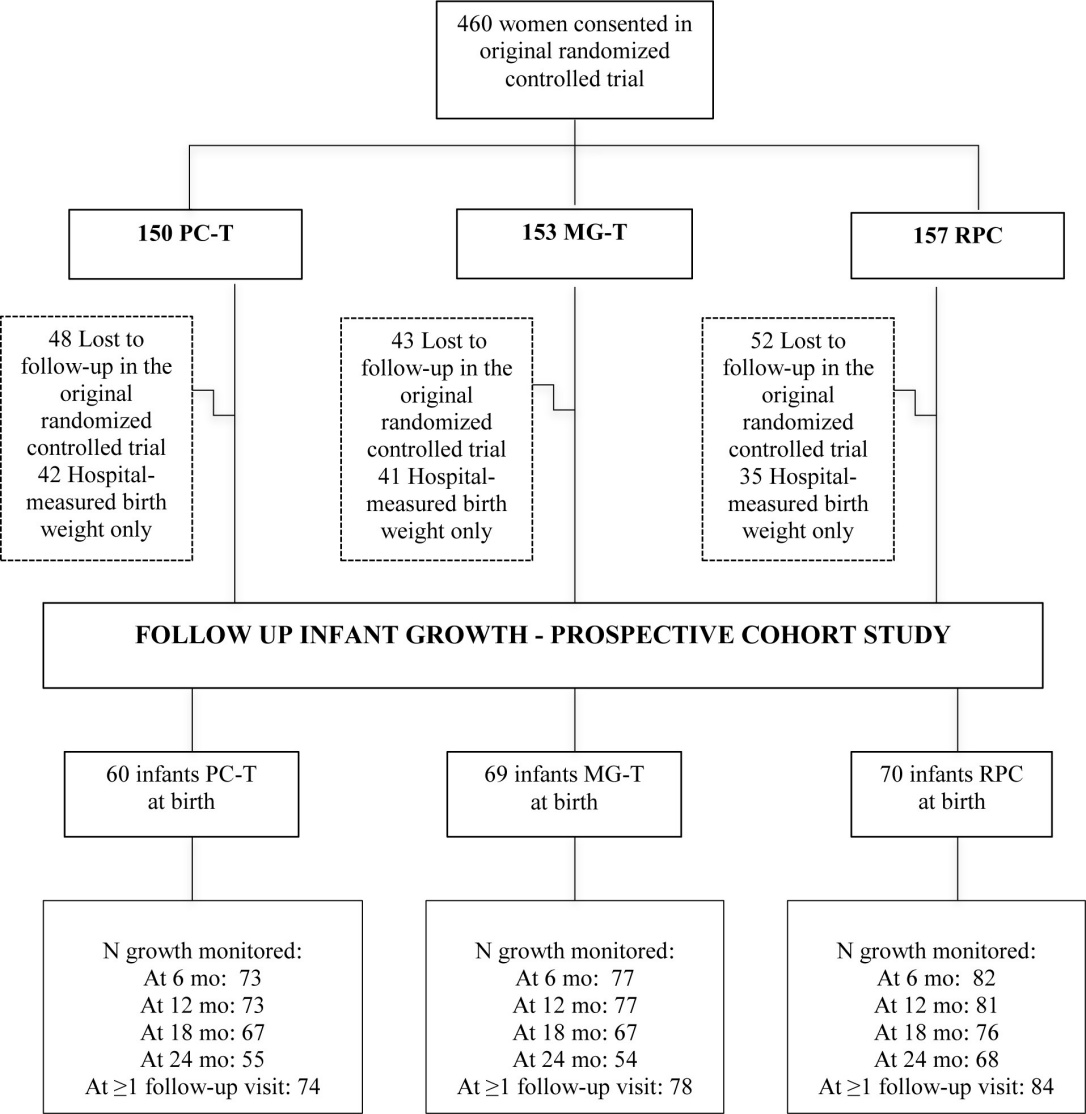
Flow diagram of participant progress throughout the VINAVAC study.

This sample size was based on a desired 80% power to detect a 135 g difference in mean birth weight between any two trial arms, which was the primary outcome of the trial. There was no additional power calculation to inform the present study, because the sample size available was based on the size of the original trial and attrition during follow-up. Of those 460 women, 317 completed the pregnancy study, and infant growth data were available from 236 women whose infants were followed until 24 months of age. The number of infants measured at each time point varied, primarily because the mothers failed to keep their appointment with the health workers (**Fig 1**). One infant in the RPC group died.

### Statistical methods

Statistical tests were conducted using Stata 14.2 (StataCorp LLC) and SPSS 24.0 (IBM SPSS Statistic 24.0). Descriptively, categorical variables are presented as percentages and the continuous variables as means and standard deviations. Comparisons by treatment group were tested using ANOVA (means) and chi-square tests (proportions). To examine effects of treatment-group assignment on infant growth outcomes (i.e., WHO Z-scores) at 6, 12, 18 and 24 months and over the entire 24-month study period, linear mixed-effects models were fitted that included fixed effects for treatment group, age, age-squared, and ageXgroup interaction and random effects for subject and age (unstructured correlation structure), as done previously [17]. Models included all recorded Z-scores from birth to 24 months. As a sensitivity check, models were also fitted with fixed-effect terms for child gender and maternal height; those results were highly similar to the unadjusted estimates.

Additionally, univariable and multivariable linear mixed-effects models were fitted to examine associations of maternal characteristics, neonatal factors, and breastfeeding practice with infant WAZ, LAZ, and WLZ over the period from 6 to 24 months of age. Maternal and infant factors were chosen for analysis based on hypothesized relationships with early infant growth but selected to avoid excess correlation among independent variables (e.g., exclusive breastfeeding ≥3 months included but not exclusive breastfeeding ≥6 months; birth weight included but not birth length). All models included fixed effects for treatment group, age, and age-squared and random effects for subject and age (unstructured correlation structure). Sensitivity checks for interaction between age and maternal or infant factors did not reveal meaningful or statistically significant interactions. Results were considered statistically significant at *p*<0.05. There was no formal adjustment for multiple hypothesis tests.

## Results

The distribution of the original cohort of 460 women entering the pregnancy RCT for this prospective cohort study of infant growth are shown in Fig 1. The characteristics of the 460 women recruited prior to conception and the characteristics of the 236 mothers whose infants participated in the postnatal growth study are shown in Table 1. At delivery, or the beginning of the infant growth study, the women were 22.5 ± 2.8 years of age. Most worked as farmers (74%) and lived with their husband’s parents (71%). Fifty-four percent had finished middle school, and 24% had college degrees. Less than 10% lived in households without a covered toilet or a well for their drinking water. On average, the women gained 7.5 kg during their pregnancy, and the infants were born at 39.2 ± 1.9 weeks gestation. These maternal characteristics were highly similar among the three dietary intervention groups.

**Table 1:** Characteristics of the mothers by treatment group.

	At Recruitment for the Pregnancy Trial				Postnatal Infant Growth Study			
	Total (N = 460)^1^	PC-T (N = 150)	MG-T (N = 153)	RPC (N = 157)	Total (N = 236)^2^	PC-T (N = 74)	MG-T (N = 78)	RPC (N = 84)
Age at baseline, year	21.6 ± 2.9	21.7 ± 3.2	21.3 ± 2.8	21.7 ± 2.7				
Age at delivery, year					22.5 ± 2.8	22.3 ± 3.0	22.5 ± 3.0	22.5 ± 2.6
Height, cm	152.7 ± 5.3	151.9 ± 5.1	153.7 ± 4.8	152.4 ± 5.8	152.7 ± 5.2	152.5 ± 4.9	153.4 ± 4.8	152.2 ± 5.7
Occupation, %								
*Farmer*	75.3	76.4	73.0	76.6	74.4	76.7	70.5	75.9
*Non-farmer*	24.7	23.6	27.0	23.4	25.6	23.3	29.5	24.1
Education, %								
*Primary school*	2.5	2.7	3.4	1.3	1.3	0.0	2.6	1.2
*Middle school*	53.8	52.7	53.0	55.6	53.7	52.8	55.3	53.0
*High school*	20.3	21.9	19.5	19.6	21.2	27.8	14.5	21.7
*College/University*	23.4	22.6	24.2	23.5	23.8	19.4	27.6	24.1
Living arrangement, %								
*With parent in law*	67.5	70.1	68.9	63.9	70.8	76.4	64.9	71.4
*With husband only*	12.4	11.5	8.6	16.8	9.4	8.3	6.5	13.1
*With parent*	20.1	18.4	22.5	19.4	19.7	15.3	28.6	15.5
Household latrine, %								
*Non/field/bush*	1.3	0.7	1.3	2.0	1.3	0.0	2.6	1.2
*Uncovered*	6.3	6.8	5.4	6.5	5.2	5.6	4.0	6.1
*Covered*	61.6	65.8	57.7	61.4	61.7	62.5	59.2	63.4
*Flush*	30.8	26.7	35.6	30.1	31.7	31.9	34.2	28.3
Drinking water source, %								
*Well water*	92.8	93.8	92.6	92.2	91.8	95.8	90.9	89.2
*Spring water*	6.3	5.5	7.4	5.9	6.9	4.2	9.1	7.2
*Rain water*	0.9	0.7	0.0	2.0	1.3	0.0	0.0	3.6
Gestational weight gain, kg					7.5 ± 3.6	7.5 ± 3.4	7.6 ± 3.0	7.3 ± 4.2
Gestational age, week					39.2 ± 1.9	39.0 ± 2.0	39.1 ± 1.8	39.5 ± 1.9

^1^ Women who consented to participate in the original trial.

^2^ Women whose infants were available for follow-up during at least one visit at age 6-months or later.

Values are means ± SDs or proportions (indicated by %).

Sample sizes for each variable vary because of item-specific missing data.

PC-T, food supplement from pre-pregnancy to term; MG-T, food supplement from mid-pregnancy to term; RPC, routine perinatal care.

The prevalence of exclusive breastfeeding and infant growth outcomes are shown in **Table 2**. None of the birth characteristics in the 236 infants in this feeding cohort differed from the characteristics of the 317 infants in the pregnancy cohort (17). Over 90% of the infants were exclusively breastfed at 1 month of age. At 3 months of age, exclusive breastfeeding had declined to 68%, and at 6 months only 6% were exclusively breastfed. However, 88% still received some breast milk at 12 months of age. Breastfeeding practices differed significantly among the three maternal intervention groups. Mothers in the MG-T group were more likely to be exclusively breastfeeding at 3 months (p = 0.02) and 97% were still giving breast milk to their infants at 12 months (p<0.001). In the entire study cohort, only 11.6% gave micronutrient supplements to their infants during the first six months. Supplement use was the lowest, 2.9%, in the MG-T group (p = 0.02).

**Table 2:** Infant breastfeeding and growth outcomes during the first 24 months by treatment group^1^.

		Total	PC-T	MG-T	RPC	p^1^
	n	(N = 236)	(N = 74)	(N = 78)	(N = 84)	
**Infant feeding practice**						
Exclusive BF at 1 mo, %	195	90.3	86.0	95.4	89.0	0.20
Exclusive BF at 3 mo, %	199	67.8	57.6^a^	80.6^b^	64.4^a^	0.02
Exclusive BF at 6 mo, %	206	6.3	7.9	8.8	2.7	0.26
Any BF at 12 mo, %	204	88.2	76.2^a^	97.1^b^	90.4^b^	<0.001
Using suppl. in first 6 mo, %	207	11.6	14.3^a^	2.9^b^	17.1^a^	0.02
**At birth**						
Male gender, %	236	47.5	50.0	44.9	47.6	0.82
Birth Weight, g	195	2977 ± 349	2938 ± 292	2959 ± 397	3029 ± 343	0.30
Birth Length, cm	193	49.0 ± 1.8	49.0 ± 1.6	49.0 ± 2.3	49.2 ± 1.5	0.72
MUAC, cm	192	10.3 ± 1.0	10.2 ± 1.0	10.3 ± 1.1	10.3 ± 0.9	0.74
HC, cm	192	33.9 ± 1.6	33.6 ± 1.6^a^	33.6 ± 1.4^a^	34.4 ± 1.6^b^	0.01
SGA for weight, %	192	15.6	12.3	17.9	16.2	0.68
LGA for weight, %	192	5.2	3.5	7.5	4.4	0.57
Low Birth weight, %	195	8.2	6.8	13.4	4.4	0.14
Preterm birth, %	235	9.8	13.7	10.3	6.0	0.26
**At 6 months**						
Weight, g	232	7219 ± 911	7212 ± 1085	7252 ± 783	7194 ± 861	0.92
Length, cm	232	64.7 ± 2.5	64.5 ± 2.7	64.9 ± 2.6	64.8 ± 2.4	0.61
Stunting, %	232	8.2	9.6	5.2	9.6	0.50
Wasting, %	232	3.0	6.9^a^	0^b^	2.4^a,b^	0.05
Underweight, %	232	3.9	9.6^a^	2.6^a,b^	0^b^	0.01
HC, cm	232	41.8 ± 1.6	41.8 ± 1.5	41.7 ± 1.6	41.9 ± 1.6	0.78
MUAC, cm	232	14.5 ± 1.3	14.7 ± 1.5	14.6 ± 1.2	14.4 ± 1.3	0.43
**At 12 months**						
Weight, g	230	8934 ± 1150	8931 ± 1358	8972 ± 1044	8902 ± 1051	0.93
Length, cm	230	72.8 ± 2.6	72.5 ± 2.9	72.9 ± 2.5	73.1 ± 2.5	0.38
Stunting, %	230	11.7	17.8	10.4	7.5	0.13
Wasting, %	230	1.3	2.7	1.3	0	0.33
Underweight, %	230	3.9	8.2	2.6	1.3	0.07
HC, cm	230	44.9 ±1.4	44.9 ± 1.4	45.0 ± 1.3	45.0 ± 1.4	0.96
MUAC, cm	230	15.1 ± 1.3	15.1 ± 1.4	15.2 ± 1.2	14.9 ± 1.3	0.34
**At 18 months**						
Weight, g	210	10044 ± 1165	10035 ± 1342	10182 ± 1199	9929 ± 950	0.43
Length, cm	210	78.8 ± 2.8	78.7 ± 2.9	78.6 ± 3.0	79.1 ± 2.6	0.52
Stunting, %	210	14.8	17.9	17.9	9.2	0.23
Wasting, %	210	1.0	1.5	0	1.3	0.62
Underweight, %	210	4.3	6.0	4.5	2.6	0.61
HC, cm	210	46.3 ± 1.4	46.2 ± 1.3	46.5 ± 1.5	46.4 ± 1.2	0.60
MUAC, cm	210	15.3 ± 1.3	15.3 ± 1.4	15.4 ± 1.2	15.2 ± 1.2	0.47
**At 24 months**						
Weight, g	177	10918 ± 1232	10947 ± 1364	10972 ± 1313	10851 ± 1056	0.85
Length, cm	177	83.8 ± 2.6	83.5 ± 2.6	83.8 ± 2.9	84.0 ± 2.4	0.52
Stunting, %	177	12.4	16.4	13.0	8.8	0.45
Wasting, %	177	2.3	1.8	0	4.4	0.26
Underweight, %	177	7.3	10.9	5.6	5.9	0.47
HC, cm	177	47.4 ± 1.8	47.2 ± 2.6	47.5 ± 1.5	47.4 ± 1.2	0.72
MUAC, cm	177	15.6 ± 1.1	15.5 ± 1.1	15.8 ± 1.1	15.6 ± 1.0	0.33

^1^ ANOVA test to compare means and chi-square test to comparing proportions over the three treatment groups; statistically significant (p<0.05) pair-wise differences denoted by different letter superscripts.

Value are means ± SDs or proportions (indicated by %).

Sample sizes for each variable vary slightly due to item-specific missing data.

PC-T, food supplement from pre-pregnancy to term; MG-T, food supplement from mid-pregnancy to term; RPC, routine perinatal care; BF, breast feeding; SGA, small for gestational age; LGA, large for gestational age; HC, head circumference; MUAC, mid-upper arm circumference.

Underweight was defined as Weight-for-age Z-score > -2 SD; Stunting was defined as Length-for-age Z-score > -2 SD; Wasting was defined as Weight-for-length Z-score >-2 SD.

In the entire group, infant birth weight averaged 2977 g and the length averaged 49 cm **(Table 2)**. These outcomes did not differ from the birth outcomes in the full pregnancy cohort (n = 317). Throughout the 24-month study, the overall prevalence of stunting, wasting, and underweight was relatively low. The prevalence of stunting increased from 8.2% at 6 months to 14.8% and 12.4% at 18 and 24 months, respectively. At 24 months, wasting and underweight, defined as weight/length or weight/age Z-scores greater than 2 SD below the mean was 4.4% or 5.9%, respectively. There were no significant differences in infant growth parameters among the three dietary intervention groups except for head circumference, which was about 0.8 cm higher (p = 0.01) in the RPC group at birth.

**Fig 2:**
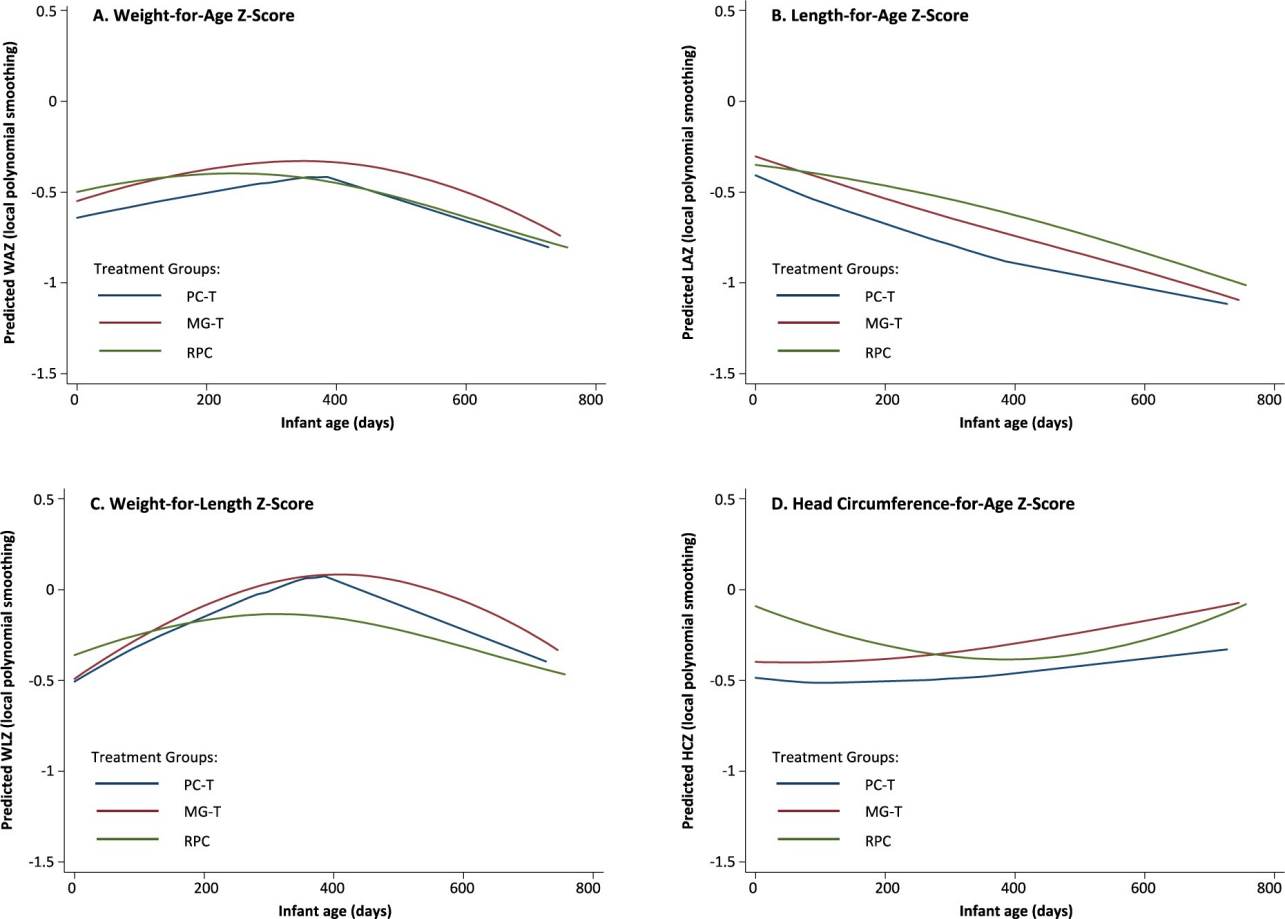
Predicted Weight-for-age (a), Length-for-age (b), Weight-for-length (c) and Head circumference (d) Z-scores from Birth to 24 Months of Age.

Linear mixed-effects models were used to examine the effects of the maternal dietary interventions on infant growth parameters (i.e., WAZ, LAZ, WLZ, and HCZ) from birth to 24 months of age **(Fig 2)**. The regression coefficients for infant anthropometry measurements are shown in **S1 Table.** Since the infants were not measured specifically at 6, 12, 18, and 24 months of age, infant age is shown as days rather than months. No meaningful differences were seen among the three diet groups. Over the study period, the PC-T group did not differ from the RPC group in WAZ (difference: -0.13; 95% CI: -0.39, 0.13), LAZ (-0.18; -0.45, 0.08), WLZ (0.01; -0.23, 0.26), or HCZ (-0.17; -0.43, 0.09). Likewise, over the study period, the MG-T group did not differ from the RPC group in WAZ (-0.02; -0.27, 0.24), LAZ (-0.06; -0.32, 0.20), WLZ (0.07; -0.17, 0.31), or HCZ (-0.03; -0.29, 0.23). Only one nominally statistically significant difference was identified: lower WLZ in the RPC group compared to the MG-T group, but only at 18 months (~540 days) and no longer evident at 24 months (~720 days). All four curves, i.e., WAZ, LAZ, WLZ, and HCZ ranged from -0.5 to 0 at 12 months (~360 days). The WAZ increased slightly in the first 360 days and then declined between days 360^t^ to 720 whereas the LAZ declined over the entire 24-month period and reached values ranging from -0.5 to -1.0 at 24 months (~720 days). At birth, the HCZ tended to be higher in the RPC group compared to the PC-T and MG-T groups (p<0.01). However, that difference was no longer evident after 12 months (~360 days).

Since the food-based supplements during gestation had a limited effect on birth outcomes and growth over the first 24 months of life, we collapsed the three groups into one larger group (n = 236) and analyzed the association between maternal characteristics and neonatal factors on infant growth as measured by WAZ, LAZ and WLZ (**Table 3**). Mother height and gestational weight gain were positively associated with the WAZ, LAZ and WLZ in the univariate regression analysis. However, when the models were adjusted for infant gender and the maternal dietary treatment in a multivariate analysis, only mother height was significantly associated with LAZ and WLZ (p<0.001). For every one cm increase in mother height, the LAZ of the infants increased by 0.05 units and the WLZ declined by 0.03 units. In the univariate analysis, for every kg increase in maternal gestational weight gain, the WAZ and LAZ increased 0.05 units (p<0.05) and WLZ increased 0.04 units (p<0.05). Infant birth weight was positively related (p<0.001) to the WAZ, LAZ, and WLZ in the multivariate model with the WAZ and WLZ increasing by about 0.1 for every 100 g increase in birth weight. LAZ only increased about 0.03 units for every 100 g increase in birth weight. Infant birth length was positively associated with the LAZ at 6, 12 and 18 months old in the univariate analysis (p<0.05). For every centimeter increase in birth length, the LAZ increased by 0.2 units at 6 months, but it declined to 0.1 units by 18 months.

**Table 3:** Associations of maternal and infant factors with infant anthropometry from 6 to 24 months of age^1^.

	**Outcome measure: weight-for-age Z-score**	
**Maternal and infant variables**	**Univariable Coefficient (95% CI)**	**Multivariable Coefficient (95% CI)**
Maternal age at delivery, y	0.02 (-0.02, 0.06)	-0.005 (-0.01, -0.00) *
Maternal height, cm	0.06 (0.04, 0.09) ***	0.001 (-0.00, 0.00)
Gestational weight gain, kg	0.05 (0.01, 0.09) **	0.0005 (-0.00, 0.01)
Lives with in-laws	0.12 (-0.15, 0.39)	0.01 (-0.02, 0.04)
Occupation is farmer	-0.40 (-0.68, -0.12) **	-0.01 (-0.04, 0.03)
Child is male	-0.25 (-0.48, -0.02) *	-0.58 (-0.61, -0.55) ***
Gestational age at birth, weeks	-0.04 (-0.10, 0.03)	0.01 (0.00, 0.02) *
Birth weight, 100g	0.08 (0.08, 0.08) ***	0.09 (0.08, 0.09) ***
Exclusive breastfeeding ≥3 months	0.11 (-0.17, 0.39)	0.004 (-0.02, 0.03)
Any breastfeeding ≥12 months	0.38 (-0.02, 0.78)	0.05 (0.01, 0.10) *
	**Outcome measure: length-for-age Z-score**	
**Maternal and infant variables**	**Univariable Coefficient (95% CI)**	**Multivariable Coefficient (95% CI)**
Maternal age at delivery, y	0.04 (-0.01, 0.08)	0.02 (-0.01, 0.06)
Maternal height, cm	0.07 (0.05, 0.09) ***	0.05 (0.03, 0.07) ***
Gestational weight gain, kg	0.05 (0.01, 0.08) *	-0.01 (-0.04, 0.02)
Lives with in-laws	0.23 (-0.02, 0.49)	0.14 (-0.07, 0.35)
Occupation is farmer	-0.41 (-0.67, -0.15) **	-0.12 (-0.34, 0.10)
Child is male	-0.19 (-0.42, 0.04)	-0.32 (-0.50, -0.14) ***
Gestational age at birth, weeks	-0.01 (-0.07, 0.05)	0.01 (-0.04, 0.06)
Birth weight, 100g	0.04 (0.03, 0.04) ***	0.03 (0.03, 0.04) ***
Exclusive breastfeeding ≥3 months	0.20 (-0.07, 0.48)	-0.05 (-0.26, 0.15)
Any breastfeeding ≥12 months	0.49 (0.10, 0.89) *	0.38 (0.07, 0.69) *
	**Outcome measure: weight-for-length Z-score**	
**Maternal and infant variables**	**Univariable Coefficient (95% CI)**	**Multivariable Coefficient (95% CI)**
Maternal age at delivery, y	0.002 (-0.04, 0.04)	-0.02 (-0.04, -0.00) *
Maternal height, cm	0.04 (0.01, 0.06) **	-0.03 (-0.05, -0.02) ***
Gestational weight gain, kg	0.04 (0.00, 0.08) *	0.01 (-0.01, 0.03)
Lives with in-laws	0.03 (-0.24, 0.29)	-0.08 (-0.22, 0.06)
Occupation is farmer	-0.25 (-0.52, 0.03)	0.05 (-0.10, 0.20)
Child is male	-0.16 (-0.39, 0.07)	-0.55 (-0.67, -0.43) ***
Gestational age at birth, weeks	-0.05 (-0.11, 0.01)	0.002 (-0.03, 0.04)
Birth weight, 100g	0.09 (0.08, 0.09) ***	0.09 (0.09, 0.10) ***
Exclusive breastfeeding ≥3 months	0.02 (-0.25, 0.28)	0.04 (-0.09, 0.18)
Any breastfeeding ≥12 months	0.18 (-0.2, 0.56)	-0.18 (-0.39, 0.03)

Statistically significant association with

*p < 0.05

**p < 0.01

***p < 0.001

^**1**^ All multivariable models additionally adjusted for group assignment in the intervention trial and child age and age-squared at the time of measurement; all models include random effects for age and infant to account for multiple observations.

## Discussion

Reducing the incidence of stunting is a primary goal of many lower- and middle-income countries (LMICs) to improve the overall health and productivity of the population. Providing adequate maternal nutrition prior to conception may improve the growth and development of the child during the first two years of life as well as pregnancy outcomes [22]. The primary goal of this research was to determine the impact of a preconception nutritional supplement and other early-life factors on pregnancy outcomes and the subsequent growth of the infant during the first two years of life. We also reported that the provision of a small (~200 kcal), nutrient-dense meal consumed five days/week from preconception to term (PC-T) or midgestation to term (MG-T) did not alter birth weight, length, or gestational age [16]. In this paper, we report the effect of the prenatal supplements before and/or during pregnancy on postnatal growth from 6 to 24 months of age. Infants born to women in the MG-T group had a higher WLZ score compared to the RPC group between 12 and 18 months of age (**Fig 2**). Also, the prevalence of wasting and underweight was significantly lower in the MG-T group compared to the PC-T group at 6 months (**Table 2**). However, by 24 months there were no significant differences in the growth indices among the three groups. We conclude, therefore, that the transient difference in infant growth observed in the MG-T group was not related to the prenatal nutrition intervention.

The small sample size of about 80 infants per group limited our ability to detect significant differences in infant growth patterns related to the prenatal intervention. A larger study of about 2500 births of women receiving a lipid-based, micronutrient supplement initiated ≥3 months prior to conception or late in the first trimester of pregnancy showed a beneficial effect of the preconception intervention on the LAZ score at birth compared to non-supplemented women [23]. It is possible that the small, 200 kcal nutritional supplement given to the PC-T and MG-T groups of women in our study reduced the amount of food available to them in their homes. Over 70% of our mothers lived with their parent in-laws where they were responsible for obtaining and preparing food for the entire family after working long hours in the field as farmers. Although we provided the supplement mid-morning at a common site outside of the home to prevent withholding any food from the mothers in the household, food deprivation may have still occurred. Also, the women generally did not eat until after the rest of the family had eaten, which may also have reduced the availability of food. In addition, the living environment of the mothers may have induced psychological, behavioral, and physiological stress that was not obviated by the small nutritional supplement provided 5 days/week. Plus, the highly-valued, first-born infant may have been favored by providing additional table food or formula even though the overall food supply in the home was limited.

The rapid economic development that occurred in rural Vietnam during the four-year study period likely influenced our results. In 2000, women of reproductive age in the Phu Tho province, where our study was done, consumed about 1500 kcal/d [24]. Ten years later, in our study, the reported energy intakes were about 33% higher [16]. Also, in 2000, 44% of the women had a BMI <18.5 whereas only 26% of our participants had a low BMI. According to World Bank statistics, poverty rates in Vietnam fell from 58% in the early 1990s to 11% in 2012 [25]. In parallel with the improved socioeconomic development, the nutritional status of the Vietnam population, and in the Phu Tho province particularly, has improved, especially among young children under five years of age. In 2005, over 25% of the children in Phu Tho were underweight; this declined to 14% in 2015. A study of under- and over-nutrition among Vietnamese children in 2011 showed an increase in overweight children, especially in urban areas [26]. However, the incidence of low weight-for-age and low height-for-age z-scores among rural children less than 2 years of age remains more common than that seen in the urban areas. Although economic growth and improved food supply for rural Vietnamese women during the past decade has improved pregnancy outcomes and infant growth, childhood undernutrition remains more prevalent in rural than in urban areas.

In addition to examining infant growth outcomes associated with the original intervention, we examined the effects of other maternal and infant factors on infant growth during the first 24 months of life (**Table 3**). In the univariate analysis, maternal height and gestational weight gain were positively associated with higher WAZ, LAZ and WLZ scores. Working as a farmer was associated with a lower WAZ and LAZ, but it was not significantly related to WLZ scores. It is interesting that working as a farmer lowered gestational weight gain without reducing birth weight [16].

In both the univariate and multivariate analyses (**Table 3),** maternal height and weight gain and birth weight were positively related to WAZ, LAZ, and WLZ scores. The association between gestational weight gain and birth weight is well established [7] but, much less is known about the relationship between gestational weight gain and infant growth patterns during the first 24 months of life. In studies from the USA, high gestational weight gains were associated with higher WAZ, LAZ, and WLZ scores during the first 3 years of age [27, 28]. But, information regarding the relationship between maternal body size, gestational weight gain and infant growth from low-income countries is limited. A recent study in rural Vietnam found that maternal height and weight were significantly related to offspring LAZ at birth and at 2 years [29]. These investigators also found that the timing of maternal gestational weight gain influenced fetal growth. Appropriate maternal weight gain during the first half of pregnancy reduced the risk of a small-for-gestational age infant by 43% [30]. In our study of pregnancy outcomes [16], maternal height was associated with gestational weight gain (p<0.01). This subsequent evaluation of growth during the first 24 months of life shows that both maternal height and gestational weight gain were associated with WAZ, LAZ, and WLZ scores. Thus, taller women tend to gain more weight s during pregnancy, and they have larger babies who grow better postnatally. However, this relationship disappears in the multivariate analysis when adjustments were made for the group assignment in the intervention trial and the child’s age at the time of measurement. Future studies are needed to evaluate the relationship between maternal height and gestational weight gain on breast milk volume and/or composition and infant growth. Possibly, improved nutrition of taller mothers alters the composition of breast milk and thereby reduces infections in the infant which increases the availability of dietary energy and nutrients for infant growth. It has been shown, for example, that providing iron, folate, and zinc to pregnant women improved the immune function in their infants and reduced diarrheal disease during the first six months of life [31].

A strength of this study is the comprehensive evaluation of maternal and neonatal factors that influence infant growth during the first 2 years of life in a low-middle income country. Most of the previous studies have focused on high gestational weight gains and excessive infant growth in high-income settings. Also, this study is unique in that about 75% of the mothers engaged in heavy physical work as farmers throughout pregnancy and lactation. Yet, nearly 70% of those women were exclusively breastfeeding at 3 months. This was possible because the fields were close to their homes and they could either return home to breastfeed the infant or carry the infant with them to the field. Thus, the data show that exclusive breastfeeding can be sustained in rural settings where the women are doing heavy physical work and consuming rice-based diets providing about 2000 kcal/d. Limitations of the study include the small sample size that reduced our ability to detect significant differences in infant growth as a result of the mother’s prenatal dietary intervention. It also would have been interesting to follow the infants for 36 months to see if the downward trend in the LAZ score over the first 24 months continued to decline or leveled off.

In conclusion, our data show that providing a nutrient-dense, food-based supplement to women prior to and/or during pregnancy did not improve infant growth during the first 2 years of life. However, maternal height and gestational weight gain was positively associated with the infant’s WAZ, LAZ and WLZ over the first 24 months. These associations disappeared when adjustments were made for the mother’s dietary treatment group and the child’s age. The data suggest, however, that food supplementation programs designed to improve gestational weight gain among women performing heavy work throughout the reproductive cycle may improve the infant’s postnatal growth.

## Supporting information

S1 Checklist CONSORT 2010 checklist of information to include when reporting a randomised trial*. (DOC)

S1 Table (CSV)

S1 File (DOC)

## Abbreviations

ASF: animal source food
BMI: body mass index
MG-T: mid-gestation to term
PC-T: pre-conception to term
RPC: routine prenatal care
SGA: small for gestational age
MUAC: mid-upper-arm circumference
WAZ: weight-for-age Z-score
LAZ: length-for-age Z-score
WLZ: weight-for-length Z-score
